# A Genome-Wide Association Study Identifies a Locus on *TERT* for Mean Telomere Length in Han Chinese

**DOI:** 10.1371/journal.pone.0085043

**Published:** 2014-01-21

**Authors:** Yun Liu, Lan Cao, Zhiqiang Li, Daizhan Zhou, Wanqing Liu, Qin Shen, Yanting Wu, Dan Zhang, Xun Hu, Ting Wang, Junyi Ye, Xiaoling Weng, Hong Zhang, Di Zhang, Zhou Zhang, Fatao Liu, Lin He, Yongyong Shi

**Affiliations:** 1 Institutes of Biomedical Sciences, Fudan University, Shanghai, PR China; 2 Bio-X Institutes, Key Laboratory for the Genetics of Developmental and Neuropsychiatric Disorders (Ministry of Education), Shanghai Jiao Tong University, Shanghai, PR China; 3 Department of Medicinal Chemistry and Molecular Pharmacology, College of Pharmacy, Purdue University, West Lafayette, Indiana, United States of America; 4 Institute for Nutritional Sciences, Shanghai Institutes for Biological Sciences, Chinese Academy of Sciences, Graduate School of the Chinese Academy of Sciences, Shanghai, PR China; 5 Women's Hospital, School of Medicine, Zhejiang University, Zhejiang, China; 6 Department of Stomatology, Ruijin Hospital Luwan Branch, Shanghai, PR China; MOE Key Laboratory of Environment and Health, School of Public Health, Tongji Medical College, Huazhong University of Science and Technology, China

## Abstract

Leukocyte telomere length (LTL) is a predictor of aging and a number of age-related diseases. We performed genome-wide association studies of mean LTL in 2632 individuals,with a two-stage replication in 3917 individuals from Chinese populations. To further validate our findings, we get the results of 696 samples from a cohort of European ancestry. We identified two loci associated with LTL that map in telomerase reverse transcriptase (*TERT*; rs2736100, *P* = 1.93×10^−5^) on chromosome 5p15.33 and near keratin 80 (*KRT80*; rs17653722, *P* = 6.96×10^−6^) on 12q13.13. In Chinese population each C allele of rs2736100 and T allele of rs17653722 was associated with a longer mean telomere length of 0.026 and 0.059 T/S, respectively, equivalent to about 3 and 7 years of average age-related telomere attrition. Our findings provide new insights into telomere regulatory mechanism and even pathogenesis of age-related diseases.

## Introduction

Telomeres are structures at the ends of eukaryotic chromosomes, which are made up of a repetitive sequence (in humans, TTAGGG) and have a major role in genomic stability. Telomere length is important in determining telomere function, whose dysregulation can lead to cell death, cell senescence, or abnormal cell proliferation [1]. In humans, leukocyte telomere length (LTL) progressively shortens with age because of the inability of DNA polymerase to fully replicate the 3′ end of the DNA strand in mitotic division, and is frequently reported to be relatively shorter in aging-related diseases: such as Alzheimer's disease [2] and vascular dementia [3]. LTL varies among individuals with the same age, and is found to be inheritable in quantitative-trait linkage analyses of sib pairs, with heritability estimates ranging from 36% to 86% [4]–[7].

In some cells, such as germ cells and proliferative stem cells that require renewal, telomere length is maintained by telomerase, a large RNA–protein complex consisting of a reverse transcriptase (TERT) and an RNA template (TERC) [8], [9]. The latter component complexes with TERT and provides the template for the synthesis of telomere repeats. Mutations in the *TERT* and *TERC* genes cause telomere shortening and are the major risk factors for rare syndromes, including idiopathic pulmonary fibrosis, aplastic anemia and dyskeratosis congenita [10]. *TERT* gene at 5p15.33 encodes the catalytic protein component of telomerase. However, a consistent conclusion could not be drawn from the association studies between *TERT* and LTL. Atzmon *et al.* reported that one haplotype of *TERT* (consisting of rs2853669, rs2736098, rs33954691 and rs2853691) was associated with LTL [11], but this association couldn't be verified by Soerensen *et al.* [12]. SNPs at 5p15.33 have been reported to be associated with risk of lung cancer in Chinese Populations [13], [14]. TERC, which serves as a template for addition of multiple 6 bp (TTAGGG) telomere repeats, is another main component of telomerase. The association between *TERC* and LTL has been identified by two recent genome-wide association studies (GWAS) [15], [16], and this association was validated in different studies including a recently report in the Han Chinese population [12], [17]. Notably, recent genome-wide studies of populations of European ancestry have revealed additional variants associated with LTL [7], [16], [18], [19], [20]. However, much of the heritability of LTL remains unaccounted for, and the pathways or biological mechanisms that underlie LTL variation are still largely unknown, e.g. *TERC* only accounts for no more than 1% of variation in telomere length [15], [16]. To identify more variants that affect telomere length, we performed GWAS analyses in one Han Chinese cohort and then replicated promising signals in two further Han Chinese cohorts.

## Results

The discovery cohort comprised 2,632 individuals (1,318 Type 2 diabetes patients and 1,312 health controls), which had been used and described in our previous study [21]. Further details of the cohort are given in Table S1. Principal-component analysis (PCA) was used to evaluate the population structure of samples of the discovery stage in comparison to the Hapmap populations, and the first two eigenvectors were plotted in Figure S1, which differentiating the Asian populations (CHB and JPT) and the study samples from the Caucasian (CEU) and Yoruba (YRI) samples clearly. LTL were approximately distributed normally (Figure S2) and showed the expected decline according to ages (Figure S3).

We analyzed the association of T/S ratio with individual's genotype in the discovery cohort, adjusted for age, gender and disease status. The quantile-quantile plots for the discovery cohort are shown in Figure S4, which showed little evidence for inflation due to population stratification (genomic inflation factor λ = 1.011). In the initial study, no SNP reached genome-wide significance (*P*<5.0×10^−8^), including previously reported SNPs on *TERC* [11] and *OBFC1* [12] (Figure S5). We set a pragmatic significance threshold of *P*<1×10^−4^ in the discovery cohort to determine which SNPs to be selected for replication study in an independent cohort comprised of 2,533 individuals (1,173 subjects with Type 2 diabetes and 1,360 healthy controls) (Rep1 cohort). Details of the Rep1 cohort are given in Table S1.

Totally, 20 SNPs were selected for replication, including one SNP rs2736100 (*P*_GWAS_ = 8.29×10^−3^), located on *TERT* (Table S2). We also z-transformed mean leukocyte telomere lengths in individual cohort to obtain comparable results and performed a meta-analysis (Table S3). We successfully replicated the association findings near genes *KRT80* and *TERT* in Rep1 cohort, and the associations in the GWAS-Rep1 reached nominal significance (*KRT80*: rs17653722, *P*_GWAS-Rep1_ = 5.85×10^−7^, p-values for the heterogeneity test: *P*_het_ = 0.463; *TERT*: rs2736100, *P*_GWAS-Rep1_ = 4.03×10^−4^, *P*_het_ = 0.942) (Table 1), and the direction of the effect was consistent with our findings in the discovery stage. And, we examined the association of these two SNPs with telomere length in additional cohort of Han Chinese (Rep2 cohort) comprised of 1,384 individuals (618 Type 2 diabetes and 766 healthy controls). As shown in Table 1, the meta-analysis results of both rs17653722 (*P*_GWAS-Rep1-Rep2_ = 6.96×10^−6^, *P*_het_ = 0.071) and rs2736100 (*P*_GWAS-Rep1-Rep2_ = 1.93×10^−5^, *P*_het_ = 0.790) still showed significant associations with telomere length. For those markers reported to have associations with LTL in previously GWAS, we only found rs12638862, which was in high linkage disequilibrium (r^2^ = 0.928 in CHB) with rs1317082 in *TERC*, showed association with LTL in our GWAS results (*P*_GWAS_ = 5.57×10^−3^, A allele β = 0.027). A full listing of SNPs is provided in Table S5.

**Table 1:** Z-score based meta-analysis results for GWAS and replication studies for two leading SNPs on 5p15.33 and 12q13.13.

				GWAS		Rep1		GWAS-Rep1-META			Rep2		GWAS-Rep1-Rep2-META		
CHR	SNP	BP	A1	Beta (SE)	*P*	Beta (SE)	*P*	Beta	*P*	*P*het	Beta (SE)	*P*	Beta	*P*	*P*het
5	rs2736100	1339516	C	0.0759 (0.0288)	8.36E-03	0.0728(0.0309)	1.85E-02	0.0744	4.03E-04	0.942	0.1071(0.0430)	1.29E-02	0.0808	1.93E-05	0.79
12	rs17653722	50873785	T	0.1731 (0.0419)	3.74E-05	0.1284(0.0442)	3.69E-03	0.1519	5.85E-07	0.463	0.0033(0.0609)	9.57E-01	0.1223	6.96E-06	0.071

Note: In each panel, markers (SNP) are given along with chromosomal (CHR) and base pair (BP) positions.

A1, minor allele. SE, standard error. Beta coefficients based on z-scores.

*P*het: p-values for the heterogeneity test.

To further validate our findings, we get the genotyping results of rs2736100 in an additional cohort of European ancestry (Rep3 cohort), including 696 samples in total (Table S1). By meta-analysis, we got significant association of rs2736100 (*P*_GWAS-Rep1-Rep2-Rep3_ = 4.45×10^−6^, *P*_het_ = 0.915), which strongly support our results.

## Discussion

**Figure 1:**
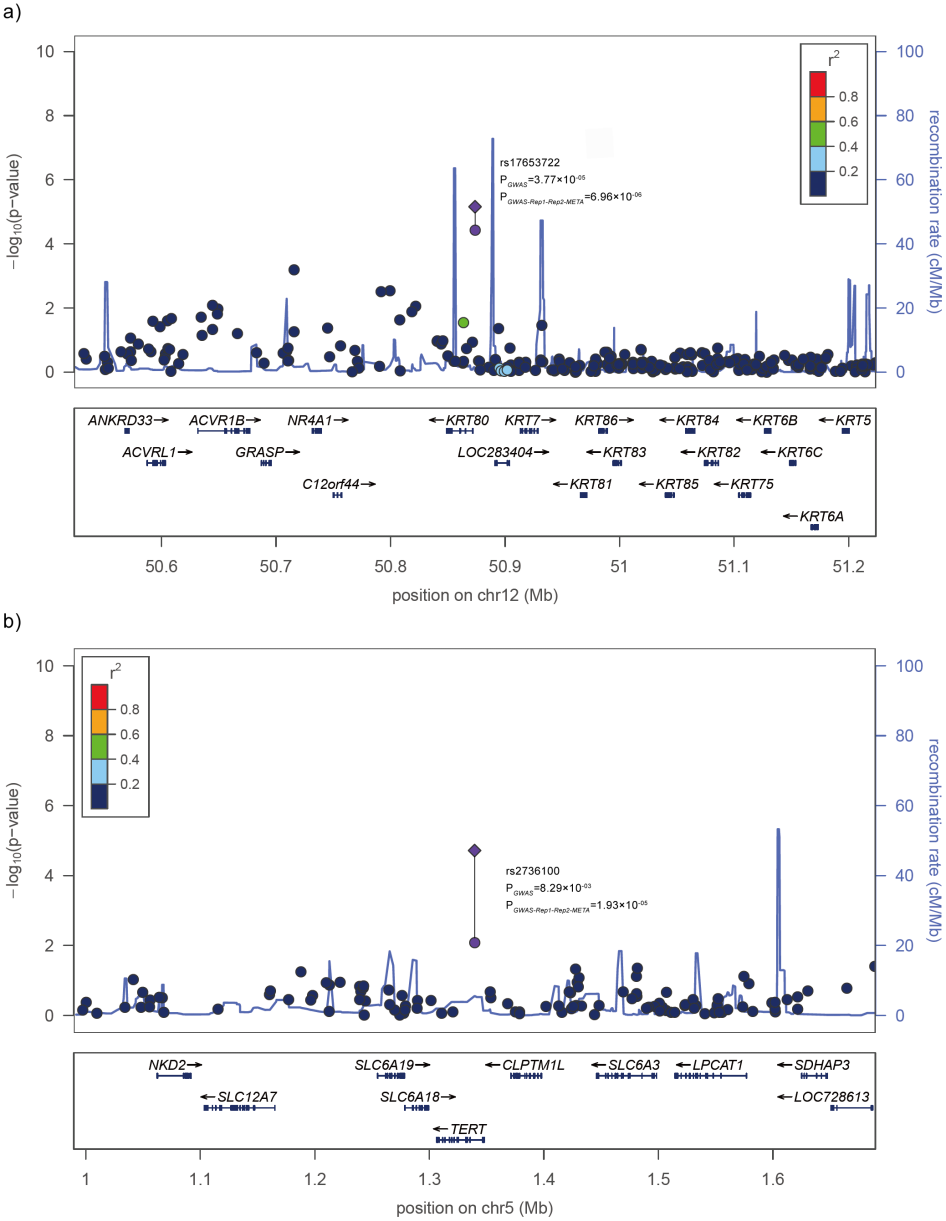
Regional plots of the two loci associated with telomere length. Results are shown for the 12q13.13 (a) and 5p15.33 (b) regions. Top, −log_10_ *P* values are shown for SNPs for the region 350 kb on either side of the marker SNPs. *P*_GWAS_ is for results obtained from the discovery stage and is shown for genotyped (circle). *P*_GWAS-Rep1-Rep2_ is for results obtained from the combination of the initial and replication study data (diamond). The marker SNP is shown in purple, and the *r* ^2^ values of the other SNPs are indicated by color. The genes within the relevant regions are annotated and shown as arrows.

Notably, rs2736100 is located in intron 2 of *TERT*, which encodes telomerase reverse transcriptase (Figure 1b), played a crucial role in protection of telomere integrity. Interestingly, rs2736100 has been reported to be associated with lung cancer risks in populations of both European descent and Asian ancestry [22]. However, it is unclear whether LTL is predictive of lung cancer risk: retrospective studies report that lung cancer patients at the time of or after diagnosis have shorter telomeres than unaffected controls [23]–[25], but prospective studies report that longer telomere length is associated with lung cancer risk including a study in China [26], [27]. We found the risk allele C in lung cancer susceptibility corresponds to a longer telomere length, which is consistent with the results that individuals with longer LTL may have an increased risk of lung cancer. Our finding suggests the *TERT* locus may influence the cancers development through variation in LTL.

To date, the *TERT* locus has been identified in several independent populations for its association with telomere length. Atzmon et al. identified a common *TERT* haplotype that was associated with telomere length, but their association analysis of common *TERT* variants showed no significant association [11]. Bojesen et al. found SNPs at the *TERT* locus were associated with telomere length and SNP rs7705526 had the largest effect [28]. However, in their study, SNP rs2736100 was not highly correlated with rs7705526 (r^2^ = 0.52), nor did it show independent associations with telomere length after adjustment for rs7705526. Codd et al. identified seven loci affecting mean telomere length including rs2736100 at 5p15.33 (*TERT*) locus [20]. We found rs2736100 at the *TERT* locus was associated with mean LTL in Chinese population, observing the association between the minor allele [C] of rs2736100 and LTL (joint *P* = 1.93×10^−5^) with an effect size and direction consistent with that of Codd et al.

On 12q13.13, rs17653722 is located ∼2 kb downstream of *KRT80*, encoding keratin 80 (Figure 1a). This protein is involved in cell differentiation, localizing near desmosomal plaques in earlier stages of differentiation but then dispersing throughout the cytoplasm in terminally differentiating cells. In expression data from the lymphoblastoid cell lines, rs17653722 is nominally associated with the expression of several genes (*ACVR1B*, *C12orf44* and *KRT80*; *P*<0.05) (Table S4);

According to the cohort used in GWAS stage, we deduced that the age-telomere declining formula for Chinese population is “(T/S ratios) = −0.0081×YEAR+1.56, R^2^ = 0.052, *P*<10^−16^)”, which indicates that LTL declines on average by 0.0081 T/S per year between the ages of 20 and 90. In Chinese population each C allele of rs2736100 and T allele of rs17653722 was associated with a longer mean telomere length of 0.026 and 0.059 T/S, respectively, equivalent to about 3 and 7 years of average age-related telomere attrition (Table S2), which showed similar effect size as *TERC*, a key component of telomere length maintaining system [17].

One limitation of this study is that the sample size was not large enough and the statistical power was relatively low to identify common variants of small effects with genome-wide significance. Another limitation is that we were unable to control more environmental factors because the information was not available. The third limitation is our study used different samples as the calibrator sample of telomere length measurement in each cohort. Therefore, we z-transformed the individual telomere length measurements in each cohort to obtain comparable results. Despite these limitations, we conducted a genome-wide association study of mean LTL in Han Chinese for the first time. In summary, we have identified common genetic variants on 5p15.33 and 12q13.13 that affect telomere length in the Han Chinese population. Several promising candidate genes, including *TERT*, are implicated in these regions. Given the importance of telomeres in cellular function and the central role of telomere length in determining telomere function, the identification of these new common genetic risk variants is the first step of elucidating the telomere regulatory mechanism and even pathogenesis of age-related diseases. And, future studies aimed at accurately mapping these regions could be fruitful.

## Materials and Methods

### Ethics Statement

The study protocols were approved by the institutional review board of the ethics committee of the Shanghai Institute for Biological Sciences and conducted according to the Declaration of Helsinki Principles.

### Participants

The characteristics details of the three cohorts are shown in Table S1.Clinical information, such as age and family history, was collected by questionnaire. Written informed consent was obtained from all subjects before enrollment.

### DNA extraction

Venous blood samples anti-coagulated with EDTA were collected from all participants. High-molecular-weight genomic DNA was prepared from venous blood using the Quick Gene 610L Automatic DNA/RNA Extraction System (Fujifilm, Tokyo, Japan) and diluted to working concentrations of 50 ng/ml for SNP chip genotyping and 10–20 ng/ml for replication genotyping.

### Leukocyte telomere length (LTL) measurement

Mean LTL was measured using a previously described modified version [29] of the quantitative real-time PCR-based assay [30]. Relative telomere length was calculated as a T/S ratio with Rnase P as a reference (ABI) for each sample. For each sample the quantity of telomere repeats and the quantity of Rnase P reference were determined in duplicate in 10 µl reactions in the same plate on an ABI-Applied Biosystems 7900 HT Thermal Cycler (Applied Biosystems).

Telomere reaction contained 1× Sybr green Taqman Gene Expression master mix (Applied Biosystems, Foster City, California), 300 nM of Tel-F, 300 nM Tel-R primers and 1 ng of template DNA. (Primers: Tel-F: 5′-CGGTTTGTTTGGGTTTGGGTTTGGGTTTGGGTTTGGGTT-3′; Tel-R: 5′-GGCTTGCCTTACCCTTACCCTTACCCTTACCCTTACCCT-3′). A commercial kit was used according to the manufacturer's instructions to estimate the expression of the RNase P gene as an internal standard (TaqManRNase P Detection Reagents Kit, Applied Biosystems), using 1× Primers and TaqMan® probe reagent, 1× TaqMan® Genotyping Master mix and 3 ng of template DNA. Cycling conditions for telomere and RNase P were 90°C incubation for 10 mins followed by either 50 cycles of 95°C for 15 sec or 60°C for 1 min.

Alongside the samples each run also contained a Calibrator sample using pooled DNA. Dilution series (0.675–5 ng in two-fold dilutions) were run for both telomere and RNase P assays to establish the linear range. Good linearity was observed across this range (R^2^>0.99). Any samples outside this range were diluted and run again. For quality control all samples were run in duplicate and the duplicate values were checked for concordance. Samples showing a CV >2% were excluded and re-run. In addition, to test reproducibility of the assay, multivariable samples were randomly chosen and run again and a high level of agreement between the T/S ratios from the two runs was observed (R^2^>0.85, *P*<0.0001).

### GWAS genotyping and quality control

The genome-wide association analysis was performed using the Affymetrix Genome-Wide Human SNP Array 6.0. Quality control (QC) filtering of the GWAS data was performed by excluding arrays with Contrast QC <0.4 from further data analysis. Genotype data were generated using the birdseed algorithm [31]. For sample filtering, arrays that generated genotypes at <95% of loci were excluded. For SNP filtering (after sample filtering), SNPs with call rates <95% in either cases or controls were removed in each geographic group. SNPs with minor allele frequency (MAF) <1% or significant deviation from Hardy-Weinberg equilibrium (HWE; *P*<0.001) in controls were also excluded. SNPs passing QC were used for further analysis. After QC filtering, there were 585,206 SNPs remaining in the discovery stage.

### SNP selection criteria and replication genotyping

We selected representative SNPs fromthe ones with *P*_GWAS_<10^−4^ for the replication study. Genotyping for the replication study was performed MGB Taqman probe assays from Applied Biosystems, using TaqMan Universal PCR Master Mix reagent kits under the guidelines. PCR plates were read on an ABI7900 system (Applied Biosystems, Foster City, CA, USA). Duplicates of randomly chosen samples were genotyped for quality control and duplicate concordance rates were 100%.

### Analysis of population substructure

Population substructure was evaluated using principal-components analysis (PCA) (Figure S1) using EIGENSTRAT software [32], [33]. Twenty components, some of which were predicted to reflect ancestry differences among subjects, were generated for each sample. Logistic regression was used to determine whether there was a significant difference in component scores between cases and controls; significant components were used as covariates in the association analysis to correct for population stratification.

### Statistical methods

Mean telomere length was considered as a quantitative trait, and expressed as a T/S ratio. Multivariable linear regression was conducted to analyze the association of the T/S ratio with SNPs, after adjusted for age, gender, and diabetes status using PLINK [34]. HWE analysis was performed using PLINK, and Haploview [35] was used to generate genome-wide P plots. Quantile-quantile plots were created using the R package, and regional plots were generated using LocusZoom (see URLs). We z-transformed the individual telomere length measurements in each cohort by subtracting the mean and division by the standard deviation and a meta-analysis using the random-effect model was carried out on the basis of the results of the three cohorts using PLINK. Heterogeneity across the data sets was evaluated using the Cochran's Q test. The meta-analysis was carried out using the Mantel-Haenszel method with a random-effects model.

### Expression quantitative trait loci (eQTL) analysis

Expression profiles were analyzed in the lymphoblastoid cell lines eQTL data sets [36], [37]. The expression data set were downloaded from the NCBI GEO database. The data setconsists of gene expression profiles generated using RNA extracted from lymphoblastoid cell lines derived from the 210 unrelated HapMap individuals from four sample groups (60 CEU, 45 CHB, 45 JPT and 60 Yoruba in Ibadan (YRI)). Expression analysis was performed using Sentrix Human-6 Expression BeadChips (Illumina). The SNP genotypes from HapMap 2 were used in the analysis. The eQTLs were tested by linear regression of normalized expression levels on SNP genotypes (coded as the number of minor alleles at each SNP: 0, 1 or 2). Analyses were conducted for each population and the combined data set.

### URLs

R, http://www.r-project.org/;

LocusZoom, http://csg.sph.umich.edu/locuszoom/;

EIGENSTRAT, http://genepath.med.harvard.edu/~reich/Software.htm;

PLINK, http://pngu.mgh.harvard.edu/~purcell/ plink/;

Haploview program, http://www.broad.mit.edu/mpg/haploview;

The International HapMap Project, http://hapmap.ncbi.nlm.nih.gov/;

NCBI GEO, http:// www.ncbi.nlm.nih.gov/geo/.

## Supporting Information

Figure S1 **The principal components analysis (PCA) of samples of discovery stage and HapMap individuals (CEU, CHB+JPT and YRI).** Samples from different geographical origin were marked by different colors.
(TIF)

Figure S2 **The distribution of LTL in four cohorts.** Figure S2 shows the distribution of LTL in the four cohorts. Telomere length was normally distributed in all cohorts.
(TIFF)

Figure S3 **Age-related LTL plot in four cohorts.** Figure S3 shows the age relationship of the T/S ratio in the four cohorts. All cohorts showed the expected decline in LTL in individuals of increasing age. Regression lines are shown in black. In the cohort used in GWAS stage, we derived an age-telomere declining formula for Chinese population as “(T/S ratios) = −0.0081×YEAR+1.56, R^2^ = 0.052, *P*<10^−16^)”, which indicates that, LTL declined on average by 0.0081 T/S per year between the ages of 20 and 90.
(TIFF)

Figure S4 **Quantile-quantile Plots of GWAS stage datasets.**
(TIF)

Figure S5 **Manhattan plot in the discovery stage.**
(TIF)

Table S1 **Descriptive statistics of our samples.**
(DOC)

Table S2 **List of top SNPs (P<1×10^−4^) associating with telomere length in the GWAS stage and the results in the replication stages.**
(DOC)

Table S3 **Z-score based meta-analysis results for GWAS and replication studies for selected SNPs.**
(DOC)

Table S4 **Expression quantitative trait loci (eQTL) analysis of rs2736100and rs17653722 for lymphoblastic cell lines of HAPMAP samples.**
(DOC)

Table S5 **The results of those markers published in previously genome wide LTL association studies in our study.**
(DOC)

## References

[1] Autexier C, Lue NF (2006) The structure and function of telomerase reverse transcriptase. Annu Rev Biochem 75: 493–517. 16756500 10.1146/annurev.biochem.75.103004.142412

[2] Panossian LA, Porter VR, Valenzuela HF, Zhu X, Reback E, et al (2003) Telomere shortening in T cells correlates with Alzheimer's disease status. Neurobiol Aging 24: 77–84. 12493553 10.1016/s0197-4580(02)00043-x

[3] von Zglinicki T, Serra V, Lorenz M, Saretzki G, Lenzen-Grossimlighaus R, et al (2000) Short telomeres in patients with vascular dementia: an indicator of low antioxidative capacity and a possible risk factor? Lab Invest 80: 1739–1747. 11092534 10.1038/labinvest.3780184

[4] Andrew T, Aviv A, Falchi M, Surdulescu GL, Gardner JP, et al (2006) Mapping genetic loci that determine leukocyte telomere length in a large sample of unselected female sibling pairs. Am J Hum Genet 78: 480–486. 16400618 10.1086/500052 PMC1380290

[5] Vasa-Nicotera M, Brouilette S, Mangino M, Thompson JR, Braund P, et al (2005) Mapping of a major locus that determines telomere length in humans. Am J Hum Genet 76: 147–151. 15520935 10.1086/426734 PMC1196417

[6] Njajou OT, Cawthon RM, Damcott CM, Wu SH, Ott S, et al (2007) Telomere length is paternally inherited and is associated with parental lifespan. Proc Natl Acad Sci U S A 104: 12135–12139. 17623782 10.1073/pnas.0702703104 PMC1924539

[7] Prescott J, Kraft P, Chasman DI, Savage SA, Mirabello L, et al (2011) Genome-wide association study of relative telomere length. PLoS One 6: e19635. 21573004 10.1371/journal.pone.0019635 PMC3091863

[8] Blackburn EH, Greider CW, Szostak JW (2006) Telomeres and telomerase: the path from maize, Tetrahymena and yeast to human cancer and aging. Nat Med 12 10: 1133–1138. 17024208 10.1038/nm1006-1133

[9] Shay JW, Wright WE (2007) Hallmarks of telomeres in ageing research. J Pathol 211: 114–123. 17200948 10.1002/path.2090

[10] Armanios M, Blackburn EH (2012) The telomere syndromes. Nat Rev Genet 13: 693–704. 22965356 10.1038/nrg3246 PMC3548426

[11] Atzmon G, Cho M, Cawthon RM, Budagov T, Katz M, et al (2010) Evolution in health and medicine Sackler colloquium: Genetic variation in human telomerase is associated with telomere length in Ashkenazi centenarians. Proc Natl Acad Sci U S A 107 Suppl 1: 1710–1717. 19915151 10.1073/pnas.0906191106 PMC2868292

[12] Soerensen M, Thinggaard M, Nygaard M, Dato S, Tan Q, et al (2012) Genetic variation in TERT and TERC and human leukocyte telomere length and longevity: a cross-sectional and longitudinal analysis. Aging cell 11: 223–227. 22136229 10.1111/j.1474-9726.2011.00775.x PMC3303949

[13] Ke J, Zhong R, Zhang T, Liu L, Rui R, et al (2013) Replication study in Chinese population and meta-analysis supports association of the 5p15.33 locus with lung cancer. PLoS One 8: e62485. 23653681 10.1371/journal.pone.0062485 PMC3641186

[14] Zhong R, Liu L, Zou L, Zhu Y, Chen W, et al (2013) Genetic variations in TERT-CLPTM1L locus are associated with risk of lung cancer in chinese population. Mol Carcinog 52 Suppl 1: 118–126. 23908149 10.1002/mc.22043

[15] Codd V, Mangino M, van der Harst P, Braund PS, Kaiser M, et al (2010) Common variants near TERC are associated with mean telomere length. Nat Genet 42: 197–199. 20139977 10.1038/ng.532 PMC3773906

[16] Levy D, Neuhausen SL, Hunt SC, Kimura M, Hwang SJ, et al (2010) Genome-wide association identifies OBFC1 as a locus involved in human leukocyte telomere biology. Proc Natl Acad Sci U S A 107: 9293–9298. 20421499 10.1073/pnas.0911494107 PMC2889047

[17] Shen Q, Zhang Z, Yu L, Cao L, Zhou D, et al (2011) Common variants near TERC are associated with leukocyte telomere length in the Chinese Han population. Eur J Hum Genet 19: 721–723. 21304559 10.1038/ejhg.2011.4 PMC3110055

[18] Gu J, Chen M, Shete S, Amos CI, Kamat A, et al (2011) A genome-wide association study identifies a locus on chromosome 14q21 as a predictor of leukocyte telomere length and as a marker of susceptibility for bladder cancer. Cancer Prev Res (Phila) 4: 514–521. 21460395 10.1158/1940-6207.CAPR-11-0063 PMC3076128

[19] Mangino M, Hwang SJ, Spector TD, Hunt SC, Kimura M, et al (2012) Genome-wide meta-analysis points to CTC1 and ZNF676 as genes regulating telomere homeostasis in humans. Hum Mol Genet 21: 5385–5394. 23001564 10.1093/hmg/dds382 PMC3510758

[20] Codd V, Nelson CP, Albrecht E, Mangino M, Deelen J, et al (2013) Identification of seven loci affecting mean telomere length and their association with disease. Nat Genet 45: 422–427. 23535734 10.1038/ng.2528 PMC4006270

[21] Liu Y, Yu L, Zhang D, Chen Z, Zhou DZ, et al (2008) Positive association between variations in CDKAL1 and type 2 diabetes in Han Chinese individuals. Diabetologia 51: 2134–2137. 18766326 10.1007/s00125-008-1141-6

[22] Hu Z, Wu C, Shi Y, Guo H, Zhao X, et al (2011) A genome-wide association study identifies two new lung cancer susceptibility loci at 13q12.12 and 22q12.2 in Han Chinese. Nat Genet 43: 792–796. 21725308 10.1038/ng.875

[23] Hosgood HD 3rd, Cawthon R, He X, Chanock S, Lan Q (2009) Genetic variation in telomere maintenance genes, telomere length, and lung cancer susceptibility. Lung Cancer 66: 157–161. 19285750 10.1016/j.lungcan.2009.02.005 PMC2783462

[24] Jang JS, Choi YY, Lee WK, Choi JE, Cha SI, et al (2008) Telomere length and the risk of lung cancer. Cancer Sci 99: 1385–1389. 18452563 10.1111/j.1349-7006.2008.00831.x PMC11158548

[25] Wu X, Amos CI, Zhu Y, Zhao H, Grossman BH, et al (2003) Telomere dysfunction: a potential cancer predisposition factor. J Natl Cancer Inst 95: 1211–1218. 12928346 10.1093/jnci/djg011

[26] Shen M, Cawthon R, Rothman N, Weinstein SJ, Virtamo J, et al (2011) A prospective study of telomere length measured by monochrome multiplex quantitative PCR and risk of lung cancer. Lung Cancer 73: 133–137. 21507503 10.1016/j.lungcan.2010.11.009 PMC3509808

[27] Lan Q, Cawthon R, Gao Y, Hu W, Hosgood HD 3rd, et al (2013) Longer telomere length in peripheral white blood cells is associated with risk of lung cancer and the rs2736100 (CLPTM1L-TERT) polymorphism in a prospective cohort study among women in China. PLoS One 8: e59230. 23555636 10.1371/journal.pone.0059230 PMC3608613

[28] Bojesen SE, Pooley KA, Johnatty SE, Beesley J, Michailidou K, et al (2013) Multiple independent variants at the TERT locus are associated with telomere length and risks of breast and ovarian cancer. Nat Genet 45: 371–384. 23535731 10.1038/ng.2566 PMC3670748

[29] Shen Q, Zhao X, Yu L, Zhang Z, Zhou D, et al (2012) Association of leukocyte telomere length with type 2 diabetes in mainland Chinese populations. J Clin Endocrinol Metab 97: 1371–1374. 22319045 10.1210/jc.2011-1562

[30] Gil ME, Coetzer TL (2004) Real-time quantitative PCR of telomere length. Mol Biotechnol 27: 169–172. 15208457 10.1385/MB:27:2:169

[31] Korn JM, Kuruvilla FG, McCarroll SA, Wysoker A, Nemesh J, et al (2008) Integrated genotype calling and association analysis of SNPs, common copy number polymorphisms and rare CNVs. Nat Genet 40: 1253–1260. 18776909 10.1038/ng.237 PMC2756534

[32] Patterson N, Price AL, Reich D (2006) Population structure and eigenanalysis. PLoS Genet 2: e190. 17194218 10.1371/journal.pgen.0020190 PMC1713260

[33] Price AL, Patterson NJ, Plenge RM, Weinblatt ME, Shadick NA, et al (2006) Principal components analysis corrects for stratification in genome-wide association studies. Nat Genet 38: 904–909. 16862161 10.1038/ng1847

[34] Purcell S, Neale B, Todd-Brown K, Thomas L, Ferreira MAR, et al (2007) PLINK: A Tool Set for Whole-Genome Association and Population-Based Linkage Analyses. The American Journal of Human Genetics 81: 559–575. 17701901 10.1086/519795 PMC1950838

[35] Barrett JC, Fry B, Maller J, Daly MJ (2005) Haploview: analysis and visualization of LD and haplotype maps. Bioinformatics 21: 263–265. 15297300 10.1093/bioinformatics/bth457

[36] Stranger BE, Forrest MS, Dunning M, Ingle CE, Beazley C, et al (2007) Relative impact of nucleotide and copy number variation on gene expression phenotypes. Science 315: 848–853. 17289997 10.1126/science.1136678 PMC2665772

[37] Stranger BE, Nica AC, Forrest MS, Dimas A, Bird CP, et al (2007) Population genomics of human gene expression. Nat Genet 39: 1217–1224. 17873874 10.1038/ng2142 PMC2683249

