# Supplementary figures and images for: A Genome-Wide Association Study Identifies a Locus on TERT for Mean Telomere Length in Han Chinese
Source: PLoS One. 2014 Jan 21;9(1):e85043. doi: 10.1371/journal.pone.0085043 (PMC3897378; doi:10.1371/journal.pone.0085043)

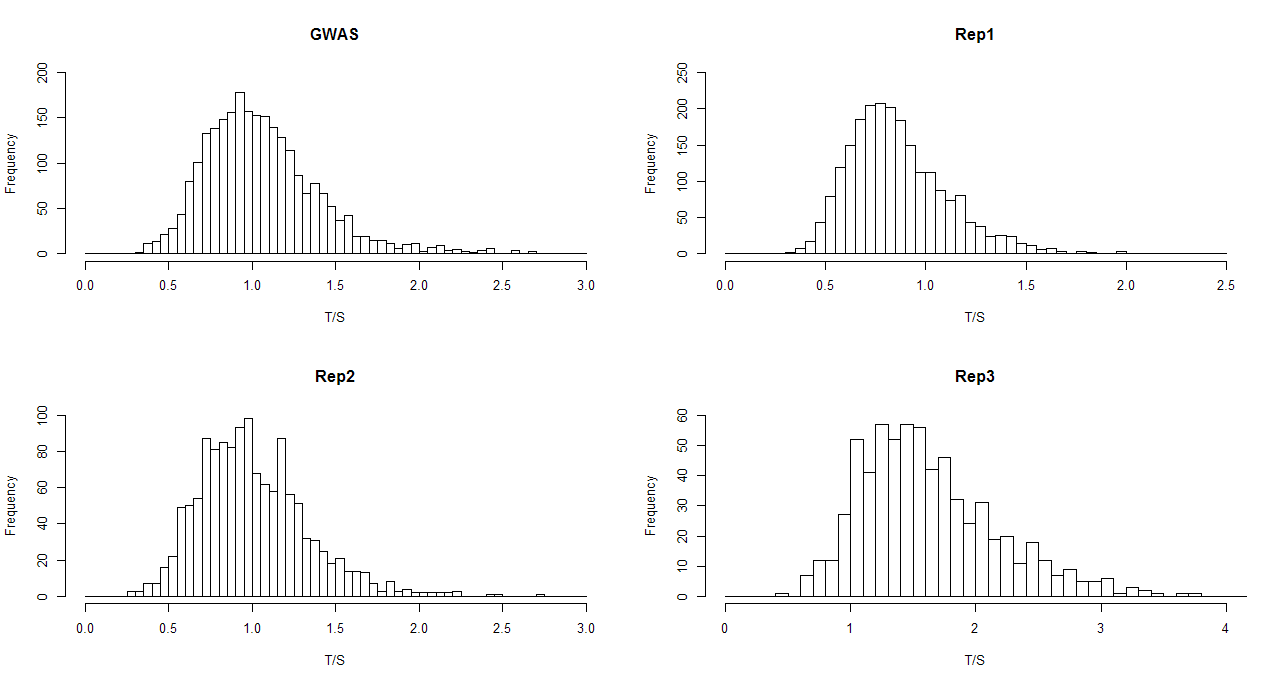

Supplement: Figure S2 — The distribution of LTL in four cohorts. Figure S2 shows the distribution of LTL in the four cohorts. Telomere length was normally distributed in all cohorts. (TIFF) [file pone.0085043.s002.tiff]

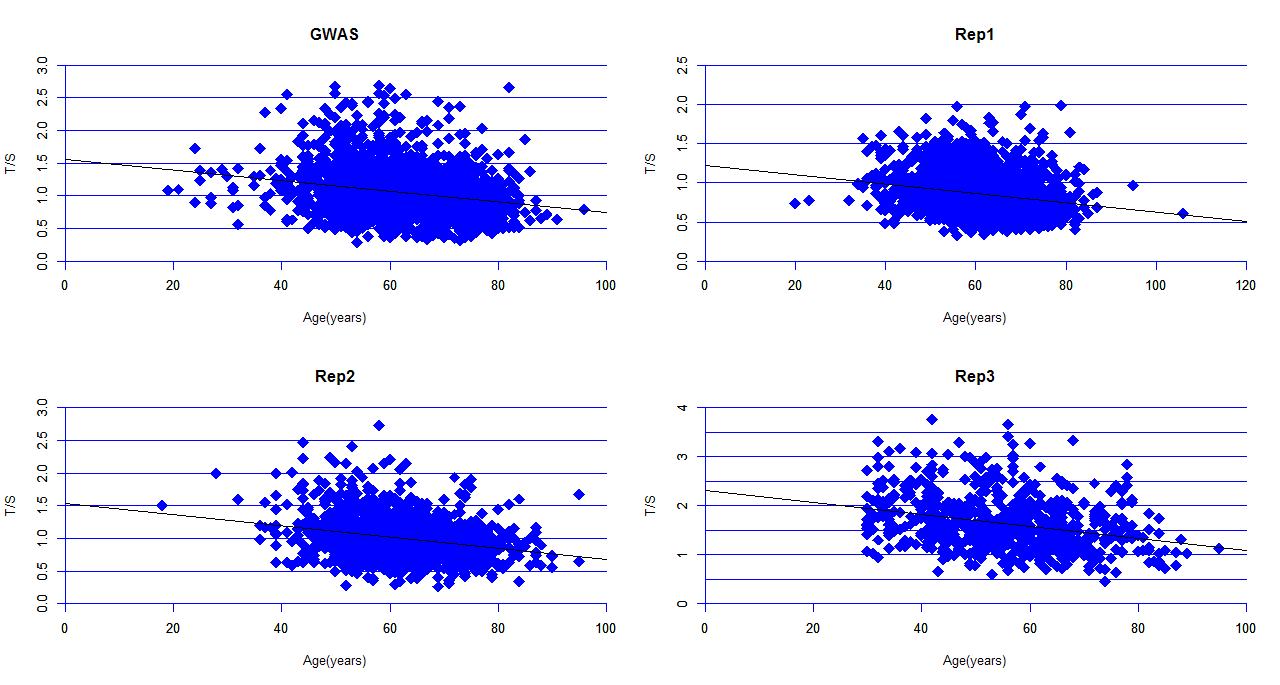

Supplement: Figure S3 — Age-related LTL plot in four cohorts. Figure S3 shows the age relationship of the T/S ratio in the four cohorts. All cohorts showed the expected decline in LTL in individuals of increasing age. Regression lines are shown in black. In the cohort used in GWAS stage, we derived an age-telomere declining formula for Chinese population as “(T/S ratios) = −0.0081×YEAR+1.56, R2 = 0.052, P<10−16)”, which indicates that, LTL declined on average by 0.0081 T/S per year between the ages of 20 and 90. (TIFF) [file pone.0085043.s003.tiff]

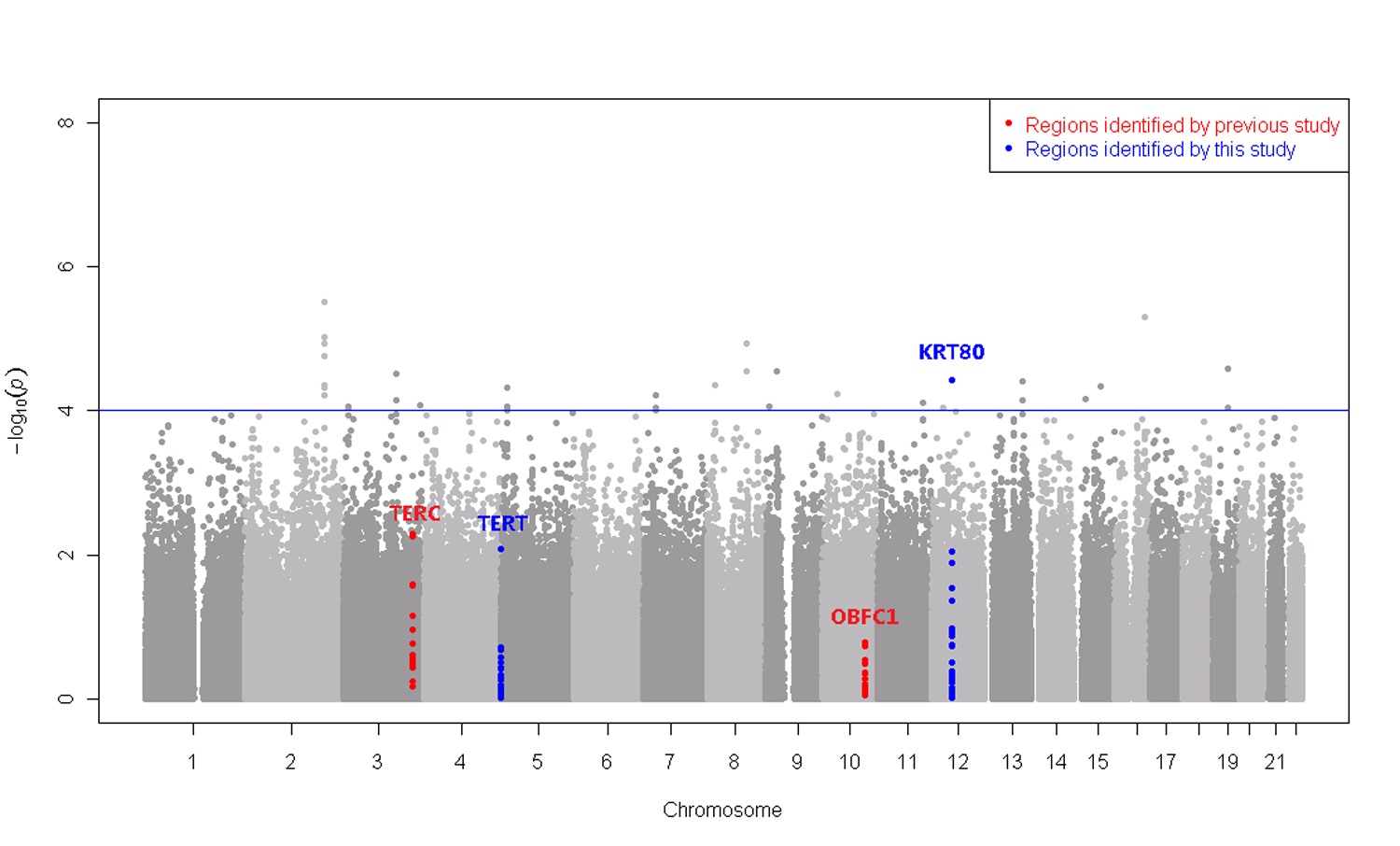

Supplement: Figure S5 — Manhattan plot in the discovery stage. (TIF) [file pone.0085043.s005.tif]
