# Supplementary material for: A Genome-Wide Association Study Identifies a Locus on TERT for Mean Telomere Length in Han Chinese
Source: PLoS One. 2014 Jan 21;9(1):e85043. doi: 10.1371/journal.pone.0085043 (PMC3897378; doi:10.1371/journal.pone.0085043)
Supplement: Table S1 — Descriptive statistics of our samples. (DOC) [file pone.0085043.s006.doc]

**Table S1.** Descriptive statistics of our samples.

GWAS cohort

|  | Non-T2D | T2D | All |
| --- | --- | --- | --- |
| Number | 1314 | 1318 | 2632 |
| Female/male | 918/395 | 772/544 | 1690/939 |
| Smoker/non-smoker | 200/1111 | 283/1029 | 483/2140 |
| Drinker/non-drinker | 198/1114 | 204/1106 | 402/2220 |
| Age (years)^a^ | 58 (53–66) | 65 (58–71) | 61(55–69) |
| BMI (kg/m2)^a^ | 24.35 (22.43–26.61) | 24.91 (22.90–27.10) | 24.62 (22.60–26.84) |
| LTL^a^ | 1.05 (0.86–1.30) | 0.97 (0.79–1.18) | 1.01(0.82–1.23) |

Abbreviations: LTL, leukocyte telomere length; T2D, type 2 diabetes.

a:Data are shown as median (25% quartile ~75% quartile).

Rep1 cohort

|  | Non-T2D | T2D | All |
| --- | --- | --- | --- |
| Number | 1360 | 1173 | 2533 |
| Female/male | 927/433 | 727/445 | 1654/878 |
| Smoker/non-smoker | 198/1110 | 185/925 | 383/2035 |
| Drinker/non-drinker | 154/1183 | 138/996 | 292/2179 |
| Age (years)^a^ | 58 (53–66) | 62 (56–70) | 60(55–68) |
| BMI (kg/m2)^a^ | 24.56 (22.48–26.67) | 25.11 (23.07–27.41) | 24.84 (22.72–27.10) |
| LTL^a^ | 0.84 (0.71–1.03) | 0.80 (0.66–0.96) | 0.82(0.69–1.00) |

Abbreviations: LTL, leukocyte telomere length; T2D, type 2 diabetes.

a:Data are shown as median (25% quartile ~75% quartile).

Rep2 cohort

|  | Non-T2D | T2D | All |
| --- | --- | --- | --- |
| Number | 766 | 618 | 1384 |
| Female/male | 512/223 | 359/245 | 871/468 |
| Smoker/non-smoker | 129/602 | 148/454 | 277/1056 |
| Drinker/non-drinker | 111/620 | 105/498 | 216/1118 |
| Age (years)^a^ | 59 (53–65) | 63 (56–71) | 61(54–68) |
| BMI (kg/m2)^a^ | 24.80 (22.52–26.89) | 25.58 (23.31–28.05) | 25.16 (22.89–27.43) |
| LTL^a^ | 0.99 (0.81–1.21) | 0.93 (0.74–1.17) | 0.96(0.77–1.19) |

Abbreviations: LTL, leukocyte telomere length; T2D, type 2 diabetes.

a:Data are shown as median (25% quartile ~75% quartile).

Rep3 cohort

|  | Rep 3 |
| --- | --- |
| Number | 696 |
| Race | White |
| Hispanic or Latine/Not Hispanic or Latine | 16/564 |
| Female/male | 331/365 |
| Age (years)^a^ | 56 (46–65) |
| LTL^a^ | 1.52 (1.23–1.94) |

Abbreviations: LTL, leukocyte telomere length.

a:Data are shown as median (25% quartile ~75% quartile).
