# Supplementary material for: A Genome-Wide Association Study Identifies a Locus on TERT for Mean Telomere Length in Han Chinese
Source: PLoS One. 2014 Jan 21;9(1):e85043. doi: 10.1371/journal.pone.0085043 (PMC3897378; doi:10.1371/journal.pone.0085043)
Supplement: Table S2 — List of top SNPs (P<1×10^−4^) associating with telomere length in the GWAS stage and the results in the replication stages. (DOC) [file pone.0085043.s007.doc]

**Table S2.** List of top SNPs (P<1x10^-4^) associating with telomere length in the GWAS stage and the results in the replication stages.

| SNP | CHR | BP | A1 | GWAS |  |  | Rep1 |  |  | Rep2 |  |  |
| --- | --- | --- | --- | --- | --- | --- | --- | --- | --- | --- | --- | --- |
|  |  |  |  | BETA | SE | *P* Value | BETA | SE | *P* Value | BETA | SE | *P* Value |
| rs13426748 | 2 | 197605805 | G | 0.056 | 0.013 | 9.54E-06 | -0.002 | 0.010 | 0.8097 |  |  |  |
| rs4256108 | 3 | 12478639 | A | 0.076 | 0.019 | 8.81E-05 | -0.006 | 0.014 | 0.6575 |  |  |  |
| rs1950079 | 3 | 133240262 | G | 0.048 | 0.012 | 3.05E-05 | 0.011 | 0.008 | 0.192 |  |  |  |
| rs10937377 | 3 | 190121372 | A | -0.085 | 0.021 | 8.26E-05 | 0.017 | 0.016 | 0.2848 |  |  |  |
| rs2736100 | 5 | 1339516 | C | 0.026 | 0.010 | 0.00829 | 0.017 | 0.007 | 0.02078 | 0.03467 | 0.01353 | 0.01053 |
| rs257778 | 5 | 15850628 | G | 0.039 | 0.010 | 9.70E-05 | -0.010 | 0.008 | 0.2061 |  |  |  |
| rs7798795 | 7 | 33940887 | C | 0.042 | 0.011 | 8.91E-05 | 0.002 | 0.008 | 0.8318 |  |  |  |
| rs6586777 | 8 | 18746751 | A | 0.064 | 0.016 | 4.40E-05 | -0.003 | 0.012 | 0.7972 |  |  |  |
| rs11784000 | 8 | 98300920 | G | -0.041 | 0.010 | 2.86E-05 | 0.012 | 0.007 | 0.1036 |  |  |  |
| rs10977362 | 9 | 8912467 | A | -0.047 | 0.012 | 8.81E-05 | -0.006 | 0.009 | 0.4949 |  |  |  |
| rs944638 | 9 | 25558851 | G | 0.070 | 0.017 | 2.87E-05 | -0.008 | 0.012 | 0.5136 |  |  |  |
| rs16937124 | 10 | 36925752 | C | 0.058 | 0.014 | 5.78E-05 | 0.017 | 0.011 | 0.1158 |  |  |  |
| rs12574578 | 11 | 112874539 | T | -0.049 | 0.012 | 7.60E-05 | 0.019 | 0.009 | 0.03579 |  |  |  |
| rs7139327 | 12 | 29536520 | T | -0.044 | 0.011 | 8.96E-05 | -0.001 | 0.009 | 0.8893 |  |  |  |
| rs17653722 | 12 | 50873785 | T | 0.0591 | 0.014 | 3.77E-05 | 0.029 | 0.011 | 6.76E-03 | 0.0025276 | 0.0193030 | 0.895847 |
| rs7337873 | 13 | 93668359 | C | -0.052 | 0.013 | 7.03E-05 | -0.006 | 0.010 | 0.4995 |  |  |  |
| rs11629663 | 15 | 27679517 | C | 0.041 | 0.010 | 6.94E-05 | 0.005 | 0.008 | 0.5446 |  |  |  |
| rs2899721 | 15 | 65036351 | G | 0.087 | 0.021 | 4.61E-05 | -0.015 | 0.016 | 0.3367 |  |  |  |
| rs406628 | 16 | 76177731 | C | 0.057 | 0.012 | 5.08E-06 | -0.003 | 0.009 | 0.7291 |  |  |  |
| rs8109247 | 19 | 38182525 | T | 0.042 | 0.011 | 9.23E-05 | -0.002 | 0.008 | 0.8178 |  |  |  |

Notes: In each panel, markers (SNP) are given along with chromosomal (CHR) and base pair (BP) positions (build36).

A1: Minor allele in our cohorts.

BETA: Effect size, a negative beta indicates a shorter mean telomere length in those carrying the minor allele.

SE: Standard error.
