# Supplementary material for: A Genome-Wide Association Study Identifies a Locus on TERT for Mean Telomere Length in Han Chinese
Source: PLoS One. 2014 Jan 21;9(1):e85043. doi: 10.1371/journal.pone.0085043 (PMC3897378; doi:10.1371/journal.pone.0085043)
Supplement: Table S3 — Z-score based meta-analysis results for GWAS and replication studies for selected SNPs. (DOC) [file pone.0085043.s008.doc]

**Table S3**. Z-score based meta-analysis results for GWAS and replication studies for selected SNPs.

| **CHR** | **SNP** | **BP** | **A1** | **GWAS** | | **Rep1** | | **GWAS-Rep1-META** | | **Rep2** | | **GWAS-Rep1-Rep2-META** | |
| --- | --- | --- | --- | --- | --- | --- | --- | --- | --- | --- | --- | --- | --- |
|  |  |  |  | **Beta (SE)** | ***P*** | **Beta (SE)** | ***P*** | **Beta** | ***P*** | **Beta (SE)** | ***P*** | **Beta** | ***P*** |
| 2 | rs13426748 | 197605805 | G | 0.1644 (0.0371) | 9.62E-06 | -0.0098 (0.0396) | 8.04E-01 | 0.0831 | 2.15E-03 |  |  |  |  |
| 3 | rs4256108 | 12478639 | A | 0.2216 (0.0564) | 8.74E-05 | -0.0138 (0.0582) | 8.13E-01 | 0.1076 | 7.89E-03 |  |  |  |  |
| 3 | rs1950079 | 133240262 | G | 0.1406 (0.0337) | 3.05E-05 | 0.0423 (0.0351) | 2.28E-01 | 0.0935 | 1.18E-04 |  |  |  |  |
| 3 | rs10937377 | 190121372 | A | -0.2477 (0.0628) | 8.27E-05 | 0.0662 (0.0673) | 3.25E-01 | -0.1015 | 2.71E-02 |  |  |  |  |
| 5 | rs2736100 | 1339516 | C | 0.0759 (0.0288) | 8.36E-03 | 0.0728 (0.0309) | 1.85E-02 | 0.0744 | 4.03E-04 | 0.1071 (0.0430) | 1.29E-02 | 0.0808 | 1.93E-05 |
| 5 | rs257778 | 15850628 | G | 0.1151 (0.0295) | 9.65E-05 | -0.0380 (0.0317) | 2.31E-01 | 0.0442 | 4.06E-02 |  |  |  |  |
| 7 | rs7798795 | 33940887 | C | 0.1237 (0.0315) | 8.87E-05 | -0.0063 (0.0328) | 8.48E-01 | 0.0613 | 6.99E-03 |  |  |  |  |
| 8 | rs6586777 | 18746751 | A | 0.1862 (0.0455) | 4.39E-05 | -0.0121 (0.0483) | 8.03E-01 | 0.0931 | 4.94E-03 |  |  |  |  |
| 8 | rs11784000 | 98300920 | G | -0.1214 (0.0290) | 2.84E-05 | 0.0454 (0.0302) | 1.33E-01 | -0.0416 | 4.68E-02 |  |  |  |  |
| 9 | rs10977362 | 8912467 | A | -0.1366 (0.0348) | 8.91E-05 | -0.0278 (0.0386) | 4.70E-01 | -0.0878 | 6.80E-04 |  |  |  |  |
| 9 | rs944638 | 25558851 | G | 0.2041 (0.0486) | 2.83E-05 | -0.0286 (0.0517) | 5.80E-01 | 0.0948 | 7.45E-03 |  |  |  |  |
| 10 | rs16937124 | 36925752 | C | 0.1700 (0.0422) | 5.83E-05 | 0.0635 (0.0437) | 1.47E-01 | 0.1186 | 9.42E-05 |  |  |  |  |
| 11 | rs12574578 | 112874539 | T | -0.1442 (0.0364) | 7.56E-05 | 0.0797 (0.0383) | 3.75E-02 | -0.0379 | 1.51E-01 |  |  |  |  |
| 12 | rs7139327 | 29536520 | T | -0.1289 (0.0328) | 8.90E-05 | 0.0062 (0.0378) | 8.69E-01 | -0.0707 | 4.33E-03 |  |  |  |  |
| 12 | rs17653722 | 50873785 | T | 0.1731 (0.0419) | 3.74E-05 | 0.1284 (0.0442) | 3.69E-03 | 0.1519 | 5.85E-07 | 0.0033 (0.0609) | 9.57E-01 | 0.1223 | 6.96E-06 |
| 13 | rs7337873 | 93668359 | C | -0.1550 (0.0389) | 7.04E-05 | -0.0284 (0.0399) | 4.76E-01 | -0.0933 | 8.16E-04 |  |  |  |  |
| 15 | rs11629663 | 27679517 | C | 0.1199 (0.0301) | 6.88E-05 | 0.0299 (0.0328) | 3.62E-01 | 0.0788 | 3.79E-04 |  |  |  |  |
| 15 | rs2899721 | 65036351 | G | 0.2540 (0.0622) | 4.57E-05 | -0.0667 (0.0661) | 3.13E-01 | 0.1033 | 2.25E-02 |  |  |  |  |
| 16 | rs406628 | 76177731 | C | 0.1669 (0.0366) | 5.23E-06 | 0.0151 (0.0390) | 6.99E-01 | 0.0959 | 3.22E-04 |  |  |  |  |
| 19 | rs8109247 | 38182525 | T | 0.1228 (0.0314) | 9.42E-05 | -0.0124 (0.0348) | 7.22E-01 | 0.0622 | 7.64E-03 |  |  |  |  |

Note: In each panel, markers (SNP) are given along with chromosomal (CHR) and base pair (BP) positions (build36).

A1, minor allele. SE, standard error. Beta coefficients based on z-scores.
