# Supplementary material for: A Genome-Wide Association Study Identifies a Locus on TERT for Mean Telomere Length in Han Chinese
Source: PLoS One. 2014 Jan 21;9(1):e85043. doi: 10.1371/journal.pone.0085043 (PMC3897378; doi:10.1371/journal.pone.0085043)
Supplement: Table S4 — Expression quantitative trait loci (eQTL) analysis of rs2736100and rs17653722 for lymphoblastic cell lines of HAPMAP samples. (DOC) [file pone.0085043.s009.doc]

**Table S4**.Expression quantitative trait loci (eQTL) analysis of rs2736100and rs17653722for lymphoblastic cell lines of HAPMAP samples.

| **SNP** | **GENE** | **PROBE** | **CHB-JPT** | | **CEU** | | **YRI** | | **HAPMAP** | |
| --- | --- | --- | --- | --- | --- | --- | --- | --- | --- | --- |
|  |  |  | **BETA** | ***P*** | **BETA** | ***P*** | **BETA** | ***P*** | **BETA** | ***P*** |
| **rs2736100** | **CLPTM1L** | GI_21359964-S | -0.020 | 7.20E-01 | -0.019 | 7.45E-01 | -0.064 | 3.95E-01 | 0.016 | 6.77E-01 |
|  | **LPCAT1** | GI_33946290-S | 0.005 | 9.32E-01 | -0.052 | 4.86E-01 | 0.028 | 7.29E-01 | -0.051 | 2.39E-01 |
|  | **NKD2** | GI_31543291-S | 0.123 | 2.54E-01 | -0.263 | **4.57E-02** | 0.115 | 4.50E-01 | -0.026 | 7.18E-01 |
|  | **SLC12A7** | GI_5730042-S | 0.194 | 9.88E-02 | 0.158 | 4.02E-01 | 0.251 | 1.18E-01 | 0.093 | 3.17E-01 |
|  | **SLC6A18** | GI_32699071-S | -0.016 | 1.98E-01 | 0.000 | 9.97E-01 | -0.025 | **3.83E-02** | -0.012 | 6.65E-02 |
|  | **SLC6A3** | GI_38194225-S | -0.015 | 2.16E-01 | -0.004 | 7.83E-01 | 0.015 | 2.43E-01 | 0.001 | 8.77E-01 |
|  | **TERT** | GI_38201699-A | -0.023 | 2.78E-01 | -0.024 | 4.16E-01 | 0.022 | 6.36E-02 | -0.007 | 5.54E-01 |
| **rs17653722** | **ACVR1B** | GI_33598913-I | -0.023 | 7.15E-01 | -0.012 | 6.47E-01 | 0.155 | **4.03E-02** | 0.018 | 5.10E-01 |
|  | **C12orf44** | GI_34222337-S | -0.093 | 1.50E-01 | -0.001 | 9.90E-01 | 0.237 | **3.47E-02** | 0.001 | 9.73E-01 |
|  | **GRASP** | GI_32171220-S | 0.068 | 2.33E-01 | -0.002 | 9.46E-01 | 0.109 | 1.21E-01 | 0.050 | 6.85E-02 |
|  | **KRT7** | GI_30089955-S | -0.010 | 8.98E-01 | -0.050 | 1.07E-01 | 0.087 | 3.35E-01 | 0.013 | 7.39E-01 |
|  | **KRT75** | GI_4758617-S | 0.012 | 5.43E-01 | -0.002 | 9.10E-01 | -0.017 | 6.74E-01 | 0.004 | 7.74E-01 |
|  | **KRT80** | GI_32698852-S | -0.004 | 8.17E-01 | -0.012 | 4.65E-01 | 0.057 | **3.65E-02** | 0.005 | 6.47E-01 |
|  | **KRT81** | GI_15431319-S | -0.009 | 5.65E-01 | -0.002 | 8.57E-01 | -0.019 | 5.04E-01 | -0.007 | 4.01E-01 |
|  | **KRT82** | GI_27477126-S | -0.004 | 8.54E-01 | -0.014 | 4.47E-01 | 0.001 | 9.67E-01 | -0.013 | 2.78E-01 |
|  | **KRT83** | GI_15431322-S | -0.012 | 6.05E-01 | 0.021 | 2.20E-01 | -0.021 | 5.29E-01 | 0.001 | 9.28E-01 |
|  | **KRT84** | GI_15431315-S | -0.018 | 3.68E-01 | -0.013 | 4.87E-01 | 0.005 | 9.08E-01 | -0.017 | 2.41E-01 |
|  | **KRT85** | GI_15431324-S | -0.016 | 2.56E-01 | -0.006 | 7.17E-01 | 0.018 | 5.33E-01 | -0.006 | 4.89E-01 |
|  | **KRT86** | GI_15431325-S | -0.007 | 9.23E-01 | -0.031 | 3.17E-01 | -0.076 | 3.72E-01 | 0.005 | 8.91E-01 |
|  | **NR4A1** | GI_27894342-I | -0.025 | 1.25E-01 | 0.005 | 7.50E-01 | 0.015 | 6.71E-01 | -0.002 | 8.16E-01 |

BETA, Regression coefficient; *P*, Asymptotic p-value for t-statistic. *P*<0.05 is demonstrated in bold.
