# Supplementary material for: A Genome-Wide Association Study Identifies a Locus on TERT for Mean Telomere Length in Han Chinese
Source: PLoS One. 2014 Jan 21;9(1):e85043. doi: 10.1371/journal.pone.0085043 (PMC3897378; doi:10.1371/journal.pone.0085043)
Supplement: Table S5 — The results of those markers published in previously genome wide LTL association studies in our study. (DOC) [file pone.0085043.s010.doc]

**Table S5**. The results of those markers published in previously genome wide LTL association studies in our study.

|  | | | | | | **Published** | | **The present study** | | | |
| --- | --- | --- | --- | --- | --- | --- | --- | --- | --- | --- | --- |
| **Study** | **Initial Sample Size** | **Replication Sample Size** | **Region** | **Reported Gene(s)** | **Strongest SNP‐Risk Allele** | **BETA** | ***P* value** | **SNP-Allele** | **BETA** | ***P* avlue** | **Pop/LD** |
| Mangino M September 25, 2012 Hum Mol Genet Genome-wide meta-analysis points to CTC1 and ZNF676 as genes regulating telomere homeostasis in humans. | 9,190 European ancestry individuals | 2,226 individuals | 10q24.33 | *OBFC1* | rs9419958-T | 0.0829 | 9.00E-11 | **-** | **-** | **-** | **-** |
|  |  |  | 19p12 | *ZNF676* | rs412658-T | 0.0497 | 1.00E-08 | rs8106346-G | 0.01 | 0.335 | CEU/0.959;  CHB/0.973 |
|  |  |  | 3q26.2 | *TERC* | rs1317082-G | 0.0679 | 1.00E-08 | rs12638862-A | 0.027 | **5.57E-3** | CEU/0.955;  CHB/0.928 |
|  |  |  | 17p13.1 | *CTC1* | rs3027234-T | 0.0573 | 2.00E-08 | rs11651993-C | 0.004 | 0.764 | CEU/0.971 |
| Prescott J May 10, 2011 PLoS One Genome-wide association study of relative telomere length. | 3,554 European ancestry individuals | 2,460 European ancestry individuals | 3q26.2 | *TERC* | rs12696304-G | 0.03 | 2.00E-14 | rs12696304-C | 0.012 | 0.244 | 1 |
| Gu J April 02, 2011 Cancer Prev Res (Phila) A genome-wide association study identifies a locus on chromosome 14q21 as a predictor of leukocyte telomere length and as a marker of susceptibility for bladder cancer. | 459 individuals | 1,160 individuals | 20q12 | *DHX35* | rs6028466-? | 0.19 | 3.00E-07 | **-** | **-** | **-** | **-** |
|  |  |  | 14q22.3 | *PELI2* | rs398652-? | 0.12 | 2.00E-06 | rs398652-G | -0.011 | 0.268 | 1 |
|  |  |  | 1p34.2 | *WDR65* | rs621559-? | 0.16 | 2.00E-06 | Rs11210811-G | -0.011 | 0.335 | CEU/1;  CHB/0. 869 |
|  |  |  | 6q22.1 | *KPNA5* | rs654128-? | 0.12 | 3.00E-06 | **-** | **-** | **-** | **-** |
| Levy D April 26, 2010 ProcNatlAcadSci U S A Genome-wide association identifies OBFC1 as a locus involved in human leukocyte telomere biology. | 3,417 European ancestry individuals | 1,893 African American and white individuals, 2,876 British women | 10q24.33 | *OBFC1* | rs4387287-A | 0.1 | 2.00E-11 | rs11191835-G | -0.009 | 0.51 | CHB/0.904 |
|  |  |  | 19p12 | *ZNF676* | rs1975174-T | 0.05 | 2.00E-06 | rs1975174-G | -0.001 | 0.89 | 1 |
|  |  |  | 2q22.1 | *CXCR4* | rs4452212-A | 0.05 | 2.00E-06 | **-** | **-** | **-** | **-** |
|  |  |  | 6p21.33 | *SLC44A4* | rs2736428-T | 0.05 | 3.00E-06 | **-** | **-** | **-** | **-** |
| Codd V February 07, 2010 Nat Genet Common variants near TERC are associated with mean telomere length. | 2,917 European individuals | 9,492 European individuals | 3q26.2 | *TERC* | rs12696304-G | 0.11 | 4.00E-14 | rs12696304-C | 0.012 | 0.244 | 1 |
| Mangino M April 08, 2009 J Med Genet A genome-wide association study identifies a novel locus on chromosome 18q12.2 influencing white cell telomere length. | 1,625 women | 1,165 individuals | 18q12.2 | *BRUNOL4, PIKC3C* | rs2162440-G | 106 (base pairs shorter) | 3.00E-06 | rs1365665-A | 0.011 | 0.348 | CEU/1;  CHB/1 |
